# Serum irisin and apelin levels and markers of atherosclerosis in patients with subclinical hypothyroidism

**DOI:** 10.20945/2359-3997000000106

**Published:** 2019-02-01

**Authors:** Hamiyet Yilmaz Yasar, Mustafa Demirpence, Ayfer Colak, Leman Yurdakul, Merve Zeytinli, Hakan Turkon, Ferhat Ekinci, Aybike Günaslan, Erdem Yasar

**Affiliations:** 1 Tepecik Research and Training Hospital Department of Endocrinology Izmir Turkey Department of Endocrinology, Tepecik Research and Training Hospital, Izmir, Turkey; 2 Tepecik Research and Training Hospital Department of Biochemistry Izmir Turkey Department of Biochemistry, Tepecik Research and Training Hospital, Izmir, Turkey; 3 Tepecik Research and Training Hospital Department of Radiology Izmir Turkey Department of Radiology, Tepecik Research and Training Hospital, Izmir, Turkey; 4 Çanakkale Onsekiz Mart University Canakkale Onsekiz Mart University Department of Biochemistry Canakkale Turkey Department of Biochemistry, Canakkale Onsekiz Mart University, Canakkale, Turkey; 5 Hatay State Hospital Department of Internal Medicine Hatay Turkey Department of Internal Medicine, Hatay State Hospital, Hatay, Turkey; 6 Ataturk Research and Training Hospital Department of Anesthesiology and Algology Izmir Turkey Department of Anesthesiology and Algology, Ataturk Research and Training Hospital, Izmir, Turkey

**Keywords:** Subcinical hypothyroidism, irisin, apelin, atherosclerosis, carotid intima-media thickness

## Abstract

**Objective:**

In this study, we aimed to evaluate serum irisin and apelin levels in patients with subclinical hypothyroidism (SCH) when they were subclinical hypothyroid and become euthyroid after levothyroxine therapy and association of these adipokines with markers of atherosclerosis such as serum homocysteine levels and carotid intima-media thickness (IMT).

**Subjects and methods:**

The study included 160 patients with newly diagnosed subclinical hypothyroidism due to Hashimoto's thyroiditis and 86 euthyroid healty subjects. Serum glucose and lipid profile, insulin, HOMA, TSH, free T3, free T4, anti-thyroperoxidase and anti-thyroglobulin antibodies, homocysteine, apelin and irisin levels were measured in all study subjects. Thyroid and carotid ultrasound examinations were performed. The subclinical hypothyroid group was reevaluated after 12-weeks of levothyroxine therapy when they became euthyroid.

**Results:**

Clinical characteristics of the patient and control group were similar. Glucose, insulin and HOMA levels, lipid parameters and free T3 were similar between the two groups.. Serum homocystein was higher and apelin was lower in patients with SCH, but irisin levels were similar between the two groups. While thyroid volume was lower, carotid IMT was significantly greater in patients with SCH (pCarotidIMT:0,01). After 12-weeks of levothyroxine therapy, all the studied parameters remained unchanged except, serum freeT4, TSH, homocystein and apelin. While homocystein decreased (p: 0,001), apelin increased significantly (p = 0,049). In multivariate analysis, low apelin levels significantly contributed to carotid IMT (p = 0,041).

**Conclusions:**

Apelin-APJ system may play a role in vascular and cardiac dysfunction in patients with SCH and treatment of this condition may improve the risk of cardiovascular disease.

## INTRODUCTION

Subclinical hypothyroidism (SCH) is defined by elevated serum levels of TSH, with normal levels of free T3 and T4. SCH prevalence ranges between 4% and 10% in the general population and between 7% and 26 % in the older population. About 2-5% of patients with SCH progress to overt hypothyroidism annually (1). Hypothyroidism and by extension SCH is associated with a significant decrease in insulin sensitivity and metabolic syndrome (2). SCH was also shown to be positively associated with coronary heart disease (CHD) events and mortality in a meta-analysis of the most relevant prospective studies (3,4).

Irisin, the most recently identified adipomyokine, is the extracellular cleaved product of fibronectin type III domain-containing 5 (FDNC5) and is regulated by peroxisome proliferator-activated receptor gamma (PPARγ) coactivator-1 alpha (PGC1α) (5). Studies have shown that FDNC5/irisin overexpression induces browning and enhances thermogenesis in white adipose tissue, contributing to improvements in glucose homeostasis and insulin resistance (5). FDNC5, which is the precursor of irisin was shown to be present in many tissues, including the thyroid tissue (5–7). Irisin was evaluated in a variety of conditions such as type II diabetes mellitus, metabolic syndrome, insulin resistance, obesity, chronic renal disease, anorexia nervosa and hypothyroidism (8). It was also reported to be associated with increased cardiometabolic risk and suggested that it might be implicated in proinflammatory and atherogenic pathways (9).

Apelin is an endogenous ligand of the G-protein coupled angiotensin-like 1 (APJ) receptor and adipose tissue is the most important source. APJ is expressed by the heart, lung, kidney, liver, adipose tissue, gastrointestinal tract, brain, adrenal glands, endothelium and plasma cells (10,11). Apelin was studied in diabetes, insulin resistance and hypertension and it was reported to be negatively associated with hypertensive heart disease (12,13). It was also shown to protect against ischaemia-reperfusion injury.

Irisin was previously studied in patients with SCH (14,15), but only Stratigou and cols. evaluated serum irisin levels after being euthyroid with levothyroxine treatment (15). Apelin was studied in patients with SCH (10,13), however it was also not evaluated after becoming euthyroid. Therefore in this study, we aimed to investigate serum irisin and apelin levels – which were reported to be associated with increased cardiometabolic risk – in patients with SCH and the effect of levothyroxine therapy on these adipokines. We also aimed to investigate the correlation of these adipokines with surrogate markers of atherosclerosis such as serum homocysteine levels and carotid intima-media thickness (IMT).

## SUBJECTS AND METHODS

### Patients and study design

The study was a single-center, prospective, case-control study including patients with SCH. A total of 160 patients attending to our outpatient Endocrinology Clinic of Tepecik Research and Training Hospital with newly diagnosed SCH due to Hashimoto's thyroiditis (age range, 20-72 years) were recruited between August 2014 and June 2015. The patients had not had yet levothyroxine treatment. The control group included 86 euthyroid healthy subjects (age range, 22-55 years) attending to our family practice outpatient clinic just for check-up. All subclinical hypothyroid subjects had autoimmune thyroiditis (Hashimto's thyroiditis) and anti-TPO antibody was positive in all of them.

The exclusion criteria included; diabetes mellitus (fasting glucose > 126 mg/dL on 2 separate occasions), hypertension (present or past antihypertensive drug use or detection of systolic pressure > 140 mmHg and/or diastolic pressure > 90 mmHg), dyslipidemia (plasma LDL-cholesterol levels > 130 mg/dL, triglyceride levels > 150 mg/dL), acute and chronic renal disease, coronary artery disease, peripheral artery disease, neurological disease or any other chronic disease or malignancy. None of the study subjects were smokers or drank alcohol. The study was approved by the medical ethics committee of the Tepecik Research and Training Hospital and all participants provided written informed consent.

All of the study subjects underwent physical examination, laboratory assessment and thyroid and carotid artery ultrasound examination. Physical examination included measurement of blood pressure and waist circumference as well as calculation of body mass index (BMI). Blood pressure was measured with the person in a seated position after a 5 minute rest with an Omron M3 HEM-7131 electronic, auscultatory blood pressure reading machine. The first reading was discarded, and the mean of the next three consecutive readings was used. Waist circumference was measured on bare skin between the tenth rib and the iliac crest in centimeters. BMI was calculated by the ratio between weight and height squared in kg/m^2^.

The subclinical hypothyroid group was reevaluated by physical examination, laboratory assessment and carotid artery ultrasound examination after 12 weeks of levothyroxine replacement therapy when they became euthyroid. Levothyroxine replacement was started with a dose of 25 µg/day and TSH was measured every 4 weeks for dose adjustment. Euthyroid state was achieved with a mean levothyroxine dose of 73.33 ± 21.70 µg/day at 12^th^ week.

### Laboratory assessments

After an overnight fast of 12 hours, venous blood was collected from the antecubital vein. Glucose concentrations were measured by a hexokinase method with the Olympus AU-2700 analyzer. Triglycerides, total cholesterol and HDL-cholesterol were measured by an enzymatic method with an Olympus AU-2700 analyzer using reagents from Olympus Diagnostics (Gmbh, Hamburg, Germany). LDL-cholesterol was calculated by the Friedewald's equation method. Insulin, free T3 (normal range: 2.5-4.4 pg/mL), free T4 (normal range: 0.54-1.24 ng/dL), TSH (normal range: 0.34-5.6 uIU/mL), anti-TPO (normal range: 0-10 IU/mL) and anti-Tg (normal range:0-50 IU/mL) levels were measured by chemiluminescent method using an Immulite 2000 otoanalyzer (Immulite XPi, Siemens, Germany). Homeostasis model assessment (HOMA) was used as a measure of insulin sensitivity using the following equation = fasting insulin (mU/L) X glucose (mmol/L)/22.5. Insulin resistance is defined as having a HOMA value > 2.7 as suggested by Matthews and cols. (16).

Serum homocysteine levels were measured by high-performance liquid chromatography (Shimadzu LC20A, Shimadzu Corp, Kyoto, Japan) based on fluorometric detection. Serum irisin and apelin concentrations were measured with an enzyme-linked immunosorbent assay (ELISA) kit according to the manufacturer's directions (Apelin; Catalog No.: 201-12-2015, Irisin; Catalog No: 201-12-5328), Sunred biological technology, Shanghai, China. The intra-assay and inter-assay coefficients of variations were < 10% and < 12% for apelin and irisin, respectively.

### Ultrasonography of the thyroid gland and carotid arteries

The thyroid and carotid ultrasound examinations were performed by a radiologist experienced in ultrasonography with a linear probe 8-13 MHz (Toshiba Aplio 300, Tokyo, Japan). Thyroid volume was calculated by the elliptical shape volume formula (0.479 X length X width X height) for each lobe (17). Carotid IMT measurements were obtained within a region free of plaque, on the far wall of the distal CCA, 2 cm proximal to the carotid bulb and on the end diastolic phase (18). In a longitudinal view of the CCA where a clearly identified double-line pattern was observed, a 10 mm length of a straight CCA far wall segment was chosen. The mean IMT was calculated by ultrasound as the mean of the computer-based lines in the selected region.

### Statistical analysis

Results are expressed as means ± SD. The patient and control groups were compared by using Student-t test. The Chi-Square test was used for nominal variables. Bazal and post-treatment values of the study group were compared by using paired samples t-test. Correlation between MPV and other parameters were assessed by Pearson's correlation analysis. Multiple regression analysis was used to identify the independent predictors that have an effect on carotid IMT. P < 0.05 was considered statistically significant. Statistical analysis was performed with SPSS 20 statistical software.

## RESULTS

Clinical characteristics of the patient and control groups are described in Table 1. No significant differences were observed between the 2 groups according to age, gender, BMI, waist circumference and blood pressure values.

**Table 1:** Clinical characteristics of the study population

	Group with SCH n: 160	Control group n: 86	P value
Age	39.58 ± 14.87	40.40 ± 10.06	0.808
Gender (M/F)	26/134	18/68	0.171
BMI (kg/m^2^)	30.22 ± 5.71	29.60 ± 6.12	0.705
Waist circumference (cm)	93.40 ± 12.13	89.47 ± 14.16	0.294
Systolic blood pressure (mmHg)	119 ± 15.8	123.6 ± 12.8	> 0.05
Diastolic blood pressure (mmHg)	84 ± 12.7	82.8 ± 8.6	> 0.05

Fasting blood glucose, insulin levels, HOMA values, lipid parameters and free T3 were similar between the 2 groups (Table 2). As expected, free T4 levels were lower and TSH, anti-TPO levels and anti-Tg levels were significantly higher (pfT4 = 0.025, pTSH < 0.001, p-anti-TPO = 0.003 and p-anti-Tg = 0.004) in the subclinical hypothyroid group (Table 2).

**Table 2:** Laboratory parameters and carotid artery Doppler ultrasound assessment of the study population

	Group with SCH (n: 160)	Control group (n: 86)	P value
Glucose (mg/dL)	92.80 ± 10.91	93.76 ± 6.83	0.704
Insulin (µU/mL)	11.00 ± 5.99	9.11 ± 4.23	0.074
Total cholesterol (mg/dL)	204.07 ± 38.00	203.08 ± 43.32	0.931
LDL-cholesterol (mg/dL)	124.88 ± 28.37	123.41 ± 32.18	0.865
HDL-cholesterol (mg/dL)	48.78 ± 14.19	53.34 ± 14.86	0.269
Triglyceride (mg/dL)	142.89 ± 90.59	137.34 ± 89.65	0.543
fT3 (pg/mL)	3.20 ± 0.41	3.35 ± 0.38	0.201
fT4 (ng/mL)	1.06 ± 0.23	1.19 ± 0.16	0.025
TSH (uIU/mL)	7.71 ± 4.08	1.82 ± 0.82	0.001
Anti-TPO (IU/mL)	142.93 ± 99.6	7.23 ± 2.93	0.003
Anti-thyroglobulin (IU/mL)	184.25 ± 49.54	44.24 ± 28.66	0.004
Thyroid volume (mL)	10.13 ± 3.39	12.29 ± 4.28	0.040
Homocystein (mMol/L)	59.32 ± 18.54	8.59 ± 4.01	0.001
Irisin (ng/mL)	23.04 ± 25.72	22.21 ± 24.34	0.801
Apelin (ng/mL)	44.55 ± 31.84	74.94 ± 63.06	0.034
Carotid IMT (mm)	0.55 ± 0.13	0.43 ± 0.19	0.008

Serum homocysteine levels were significantly higher in patients with SCH with respect to the control group (p_homocysteine_ = 0.001). Despite similar irisin levels, apelin levels were significantly lower in patients with SCH (p_apelin_ = 0.034). Although thyroid volume was significantly lower, carotid IMT was significantly greater in patients with SCH (p thyroid volume = 0.04, p carotid IMT = 0.008).

The subclinical hypothyroid group was reevaluated with the same laboratory parameters after 12 weeks of levothyroxine replacement therapy when they became euthyroid. The basal and post-treatment data are reported in Table 3. Basal and post-treatment BMI and waist circumference measurements were similar. No significant differences were observed between basal and post-treatment serum glucose, insulin and HOMA values and lipid parameters. As expected, serum freeT4 levels were increased and TSH levels were decreased significantly (pfT4 = 0.022), Ptsh = 0.001). Serum homocysteine levels were decreased significantly from 59.53 ± 26.28 to 8.59 ± 4.01 after 12 weeks of levothyroxine treatment (p = 0.001). Although serum irisin levels remained similar, apelin levels were significantly increased from 44.55 ± 31.84 to 72.91 ± 81.67 (p = 0.035). In addition, carotid IMT decreased significantly after treatment (p carotid IMT = 0.030).

**Table 3:** Basal and 12^th^ week clinical and laboratory evaluation of patients with SCH

	Group with SCH (n: 160) Basal	Group with SCH (n: 160) after LT4 treatment	P values
BMI (kg/m^2^)	30.24 ± 5.57	29.72 ± 5.78	0.114
Waist circumference (cm)	91.68 ± 12.13	90.13 ± 12.67	0.216
Glucose (mg/dL)	92.80 ± 10.91	94.43 ± 9.48	0.273
Insulin (µU/mL)	11.00 ± 5.99	14.39 ± 8.23	0.188
Total cholesterol (mg/dL)	214.07 ± 38.00	205.85 ± 36.52	0.269
LDL-cholesterol (mg/dL)	134.88 ± 28.37	130.11 ± 30.22	0.606
HDL-cholesterol (mg/dL)	48.78 ± 14.19	46.25 ± 13.99	0.084
Triglyceride (mg/dL)	152.89 ± 90.59	158.107 ± 92.00	0.759
fT3 (pg/mL)	3.20 ± 0.41	3.16 ± 0.37	0.234
fT4 (ng/mL)	1.06 ± 0.23	1.27 ± 0.49	0.022
TSH (uIU/mL)	7.71 ± 4.08	3.51 ± 1.91	0.001
Anti-TPO (IU/mL)	142.93 ± 99.6	147.66 ± 98.14	0.154
Anti-thyroglobulin (IU/mL)	184.25 ± 49.54	143.04 ± 59.68	0.190
Homocystein (mMol/L)	59.53 ± 26.28	8.59 ± 4.01	0.001
Irisin (ng/mL)	23.04 ± 25.72	23.20 ± 24.63	0.821
Apelin (ng/mL)	44.55 ± 31.84	72.91 ± 81.67	0.035
Carotid IMT (mm)	0.55 ± 0.13	0.44 ± 0.17	0.030

**Figure 1:**
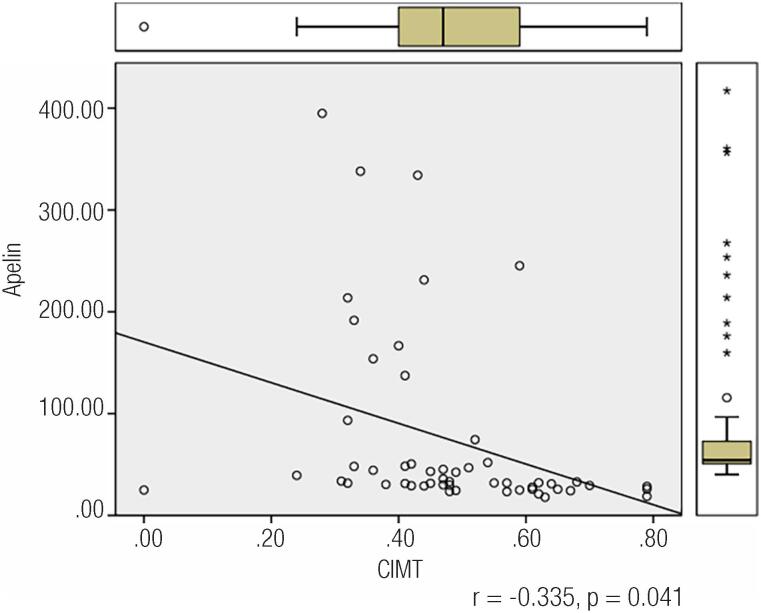
Correlation analysis between apelin levels and CIMT.

Correlation analyses between irisin, apelin, homocysteine, carotid IMT and all the other parameters studied were conducted. Irisin was negatively correlated with age (r = −0.271, p = 0.040) and apelin (r = −0.547, p = 0.001) was positively correlated with carotid IMT (r = 0.614, p = 0.001). Apelin level was negatively correlated with age (r = −0.328, p = 0.012) and carotid IMT (r = −0,611, p = 0.001), in addition to irisin. No correlation was observed between homocysteine level and other parameters. Multiple regression analysis revealed that although low apelin level significantly contributed to carotid IMT (p = 0,041) (Figure 1), high serum irisin level did not significantly affect carotid IMT (p = 0.096).

## DISCUSSION

The association of SCH with all components of metabolic syndrome and risk of cardiovascular disease have been well-studied (2–4). However the data are limited about the level of adipokines such as irisin and apelin – whose metabolic and cardiovascular effects were supposed to be similar to the effects of hypothyroidism – in patients with SCH. Therefore, in this study we investigated serum irisin and apelin levels and known cardiovascular risk factors such as serum homocysteine levels and carotid IMT, in patients with SCH. We also re-evaluated all of these parameters after 12-weeks of levothyroxine therapy when euthyroidism was achieved.

SCH has been associated with functional cardiac abnormalities, vascular abnormalities, (eg increased vascular resistance, arterial stiffness and endothelial dysfunction), and atherosclerosis (19). Several cohort studies have analyzed the association between SCH and CHD. Although in the Whickham survey, CHD events were increased in individuals with SCH, in another survey including 3,233 individuals no significant difference was observed in the prevalence of CHD between SCH patients and euthyroid controls (20,21). Likewise surrogate indexes of atherosclerosis such as serum homocysteine levels and carotid IMT have been well studied in SCH and after restoration of the euthyroid state, but the results are contradictory (22–25). Similar to some of the previous reports, we observed hyperhomocysteinemia (24,25) and significantly increased carotid IMT in subclinical hypothyroid patients (24). Also, in our study we observed significantly decreased homocysteine values with the restoration of euthyroid state after 12-weeks of levothyroxine treatment similar to Sengül and cols. (25). Yet it was reported as unchanged in other studies (23).

Irisin has a role in the browning of subcutaneous adipocytes via elevation of uncoupling protein-1 (UCP-1)and it has been shown to increase energy expenditure. Although there are conflicting results about irisin levels in type II diabetes, a meta-analysis showed that irisin levels were decreased in type II diabetes and they were found to be increased in insulin resistance and metabolic syndrome (26). Irisin was previously studied in hypothyroid patients. Ates and cols. reported increased irisin levels in hypothyroid patients and suggested that increased TSH levels might cause increased irisin levels. It was proposed that increased TSH might lead to increased adipogenesis and hormones such as leptin, ghrelin and irisin could be synthesized to keep the fat distribution in balance in the increased white adipose tissue (6). Zybek-Kocik and cols. found decreased irisin levels in long-standing hypothyroidism and suggested that it might result from muscle damage due to prolonged myopathy and leakage of irisin from damaged muscle cells (7). Only two studies evaluated circulating irisin levels in SCH: Although irisin levels were similar between euthyroid and subclinical hypothyroid patients in one study (14), it was reported to be increased in the other study (15). Stratigou and cols. suggested that irisin might represent an adipo-myokine counterbalancing the deterioration of lipid metabolism and insulin sensitivity in SCH as well as reflecting a protective compensatory mechanism against oxidative muscle and thyroid cell stress (15). In our study irisin levels in subclinical hypothyroid patients were similar to healthy controls. This may be because in Stratigou's study insulin and lipid levels were significantly higher in patients with SCH compared to the control group. In our study, these parameters were similar between the patient and control group. Also, Stratigou and cols, observed no change in serum irisin levels after 6 months of levothyroxine treatment with restoration of euthyroidism. It was suggested that this might be attributed to 1) the small number of treated patients, 2) the short follow-up period, and 3) the variation of irisin not depending solely on TSH levels. Likewise, we found similar irisin levels after treatment.

Apelin, an adipocytokine is a constituent of adipose tissue and is secreted by adipocytes that are stimulated by insulin. It is also an endogenous peptide that is a ligand for the APJ receptor and produced by endothelial cells in many parts of the body and the sites of receptor expression are linked to the different functions of apelin (13). It is a powerful inotrope and peripheral vasodilator and may be involved in fluid homeostasis, by its antidiuretic effect (27). Apelin levels were found to be increased in type II diabetes and hyperinsulinemia-dependent obesity and decreased in hypertension and hypertensive heart disease (10). In addition a more recent study suggested that the apelin-APJ system might protect the myocardium from ischemia reperfusion injury by its actions on the reperfusion injury salvage kinase pathway (13). A small amount of evidence shows that the apelin-APJ system may be involved in modulating endothelial oxidative stress and the formation of coronary atherosclerotic plaques (27,28).

Two studies have evaluated apelin levels in patients with SCH. In both studies no significant difference was observed in serum apelin levels between patients with SCH and healthy control subjects (10,13). The authors explained that these results were because, BMI was similar in both groups. In contrast to these findings, we observed significantly lower serum apelin levels in patients with SCH. Being different from these studies, we also evaluated apelin levels after becoming euthyroid with levothyroxine treatment in patients with SCH and we found a significant increase in serum apelin levels. This may be explained by SCH possibly being associated with increased risk of atherosclerosis (3–5) and treating SCH might lead to improvement in the atherosclerosis risk as suggested by changes in established markers of atherosclerosis such as homocysteine levels and carotid IMT.

In the present study, we also investigated serum homocysteine levels and carotid IMT in patients with SCH and the association of serum irisin and apelin levels with these markers of atherosclerosis. We found significant association of low apelin levels with carotid IMT.

To our knowledge, this is the first study that has evaluated serum irisin and apelin levels in patients with SCH when they were subclinical hypothyroid and had become euthyroid after levothyroxine replacement therapy. Nevertheless, our study has some limitations: the studied groups were small and the percentage of male participants was low.

In conclusion, the apelin-APJ system may play a role in vascular and cardiac dysfunction described in patients with SCH and treatment of this condition may decrease the risk of cardiovascular disease. However, further studies should be carried out to evaluate the effect of the apelin-APJ system and thyroid hormone replacement in preventing cardiovascular events and mortality.
